# Robotic-Assisted Versus Fluoroscopic-Guided Surgery on the Accuracy of Spine Pedicle Screw Placement: A Systematic Review and Meta-Analysis

**DOI:** 10.7759/cureus.54969

**Published:** 2024-02-26

**Authors:** Bongseok Jung, Justin Han, Shaya Shahsavarani, Anas M Abbas, Alexandra C Echevarria, Robert E Carrier, Alex Ngan, Austen D Katz, David Essig, Rohit Verma

**Affiliations:** 1 Orthopedic Spine Surgery, Northwell Health, Manhasset, USA; 2 Orthopedics, Donald and Barbara Zucker School of Medicine, Hempstead, USA

**Keywords:** pedicle screw, spine surgery, accuracy, robotic, robotic assistance, fluoroscopy, radiation, meta-analysis

## Abstract

Spinal fusion is a common method by which surgeons decrease instability and deformity of the spinal segment targeted. Pedicle screws are vital tools in fusion surgeries and advancements in technology have introduced several modalities of screw placement. Our objective was to evaluate the accuracy of pedicle screw placement in robot-assisted (RA) versus fluoroscopic-guided (FG) techniques. The PubMed and Cochrane Library databases were systematically reviewed from January 2007 through to August 8, 2022, to identify relevant studies. The accuracy of pedicle screw placement was determined using the Gertzbein-Robbins (GR) classification system. Facet joint violation (FJV), total case radiation dosage, total case radiation time, total operating room (OR) time, and total case blood loss were collected. Twenty-one articles fulfilled the inclusion criteria. Successful screw accuracy (GR Grade A or B) was found to be 1.02 (95% confidence interval: 1.01 - 1.04) times more likely with the RA technique. In defining accuracy solely based on the GR Grade A criteria, screws placed with RA were 1.10 (95% confidence interval: 1.06 - 1.15) times more likely to be accurate. There was no significant difference between the two techniques with respect to blood loss (Hedges' g: 1.16, 95% confidence interval: -0.75 to 3.06) or case radiation time (Hedges' g: -0.34, 95% CI: -1.22 to 0.53). FG techniques were associated with shorter operating room times (Hedges' g: -1.03, 95% confidence interval: -1.76 to -0.31), and higher case radiation dosage (Hedges' g: 1.61, 95% confidence interval: 1.11 to 2.10). This review suggests that RA may slightly increase pedicle screw accuracy and decrease per-case radiation dosage compared to FG techniques. However, total operating times for RA cases are greater than those for FG cases.

## Introduction and background

Spine surgery often requires specific utilization of equipment tailored to the procedure in question, and instrumented spinal fusion surgeries are no exception. Pedicle screws in particular are a mainstay of spinal fusion. These screws allow the surgeon to instrument multiple spinal levels and thus achieve immobilization and fusion [1]. Ultimately, this may serve multiple purposes including reduction in patient pain, deformity correction, and increased stability.

Pedicle screws have revolutionized spinal surgery since their introduction to clinical application [2]. The success of robust fixation relies on the accurate insertion of screws inside the pedicle. However, the procedure for accurate pedicle screw placement can be challenging because of the complex anatomical structures surrounding the spine and widely morphologic variations of individual pedicles [1,3-5]. Both freehand (FH) and fluoroscopy-guided (FG) techniques involve using anatomical landmarks to identify the correct entry point for the pedicle screw placement and confirming the site using lateral FG as well as a K-wire. Once this is accomplished, an anteroposterior fluoroscopy image of the site is developed to verify appropriate alignment and technique. This approach largely relies on the surgeon’s experience with tactile feel and knowledge of anatomical variations [6-8]. In contrast, the conventional approach to pedicle screw insertion does not use guidance but relies on anatomic landmarks to identify the correct site for placement of the screw. A well-documented shortcoming of this approach is the risk of screw misplacement which cannot be neglected when weighing surgical options [6,7]. A meta-analysis reported the percentage of misplaced screws to be between 5% and 25% for more than half of the included studies utilizing true FH technique while the FG technique was shown to decrease this rate to 3% to 15% [6]. Misplaced screws may have several devastating ramifications including vascular, neurological, or visceral injuries, though these outcomes are relatively rare [7].

Another innovative technique developed to improve surgeon precision and accuracy is computed tomography (CT)-navigated screw insertion. Surgical navigation as we know it today involves using marker spheres on instruments as well as specific areas of the surgical site in order to give the surgeon the ability to understand where the instrument is in relation to sensitive structures and the target area of surgery [8]. Finally, to further minimize human error, in 2004, robotics were introduced to assist in pedicle screw insertion. Mazor Robotics, an Israeli robotics company, developed a spine-mounted robot called the Mazor SpineAssist that would guide the trajectory of the instrumentation and screw placement [9]. In the years since the introduction of the SpineAssist, several other companies have joined the fray, and many improvements and innovations have been made [10].

A number of robots, and updates to prior robots, have entered the market since that time. These include the ExcelsiusGPS, TiRobot, and the ROSA spine [11]. Each robot, with its own user interface and surgical workflow, represents an enormous effort to augment the surgeon’s ability to accurately place screws. This begs the question of whether or not robots have truly yielded desirable outcomes in aspects such as accuracy of placement, intraoperative time, and radiation exposure.

In general, the spine robot’s workflow involves pre-surgical imaging, mounting of a bridge to orient the robot in space with respect to the patient’s body, intraoperative FG or CT, access to the pedicle in question (either through minimally invasive or open techniques), pedicle entry, tapping, and screw implantation using navigation [12]. Of note, the pre-surgical imaging is done prior to the day of surgery in order to maximize the efficiency of the surgeon’s preoperative planning; such imaging is also done for FH surgeries. The intraoperative imaging differs in comparison to other techniques due to the fact that each pedicle screw is assessed during and after its placement for accuracy. The overarching goal of robotic assistance (RA) is screw placement with increased accuracy and decreased operation time.

Though the literature presents many studies and reviews comparing pedicle screw placement via traditional methods to robot-guided methods, few reviews demonstrate a temporal breadth specifically comparing robotic accuracy and performance to FG without navigation. In this study, we aim to study accuracy, operative lengths, and radiation exposure in robot-guided cases to better characterize the efficacy of RA pedicle screw placement in comparison to FG pedicle screw placement.

## Review

Review protocol

This systematic review was synthesized in accordance with the Preferred Reporting Items for Systematic Reviews and Meta-Analysis (PRISMA) guidelines.

Search strategy

A comprehensive literature search of PubMed and Cochrane Central Register of Controlled Trials was undertaken for relevant published articles with no date restrictions imposed. Publications were searched using the terms of “robot pedicle screw”, "pedicle screw with robot", "RA versus FG pedicle screw", and "robot versus FG pedicle screw". The boolean operator "and" was used to search "robot and pedicle screw" and "RA and FG pedicle screw". All reference lists of the retrieved studies were analyzed for additional relevant literature. Afterwards, the authors performed a full-text review of the relevant literature. Although there was no language restriction, non-English articles were excluded from the study. Authors of relevant studies were not contacted in this process for original data. Literature was searched from January 2007 to August 8, 2022. 

Inclusion criteria

Studies that met the following inclusion criteria were included in this review: (1) those evaluating the patient population of interest: patients of all ages undergoing a robotic pedicle screw placement procedure; (2) with the following study designs: retrospective case-controlled analyses utilizing prospectively collected data, retrospective case-controlled studies, prospective randomized controlled trials (RCTs); and (3) comparing the following treatment groups: RA vs FG without navigation screw placement. 

Quality assessment

The principles of Grading of Recommendations Assessment, Development and Evaluation (GRADE) criteria were incorporated to interpret the quality of evidence from each study [13]. According to these criteria, each study's evidence was evaluated and ranked from very low, low, moderate, and high levels of certainty. Per the criteria, randomized control trials were initially rated "high"-quality evidence and the level of confidence was adjusted based on factors such as study limitations, inconsistency of results, indirectness of evidence, imprecision, and reporting bias. Observational studies were rated as "low" quality evidence which was adjusted based on factors such as the size of the study and degree of bias. All reviewers agreed on the quality of evidence from each study. Any disagreements were resolved after further consideration. 

Exclusion criteria

Unpublished abstracts and studies were excluded from this systematic review. No gray literature, defined as literature outside traditional academic publications, was included in this review. Studies that demonstrated author conflict of interest due to monetary compensation or corporate consultation were excluded from this review. Studies that utilized true FH (i.e. not utilizing intraoperative FG) as the control group were excluded. Studies whose full manuscripts were not published fully in English were excluded. Meta-analyses and other systematic reviews were excluded from the analysis. Studies that did not include exact data such as the number of screws and standard deviations were excluded. Animal studies, correspondence or letters, articles with incomplete clinical information, and articles not available in full text were excluded. Studies that utilized criteria other than the Gertzbein-Robbins (GR) classification were excluded from the accuracy analysis though they may have been included in other secondary analyses if they met other inclusion criteria.

Selection of studies

**Figure 1:**
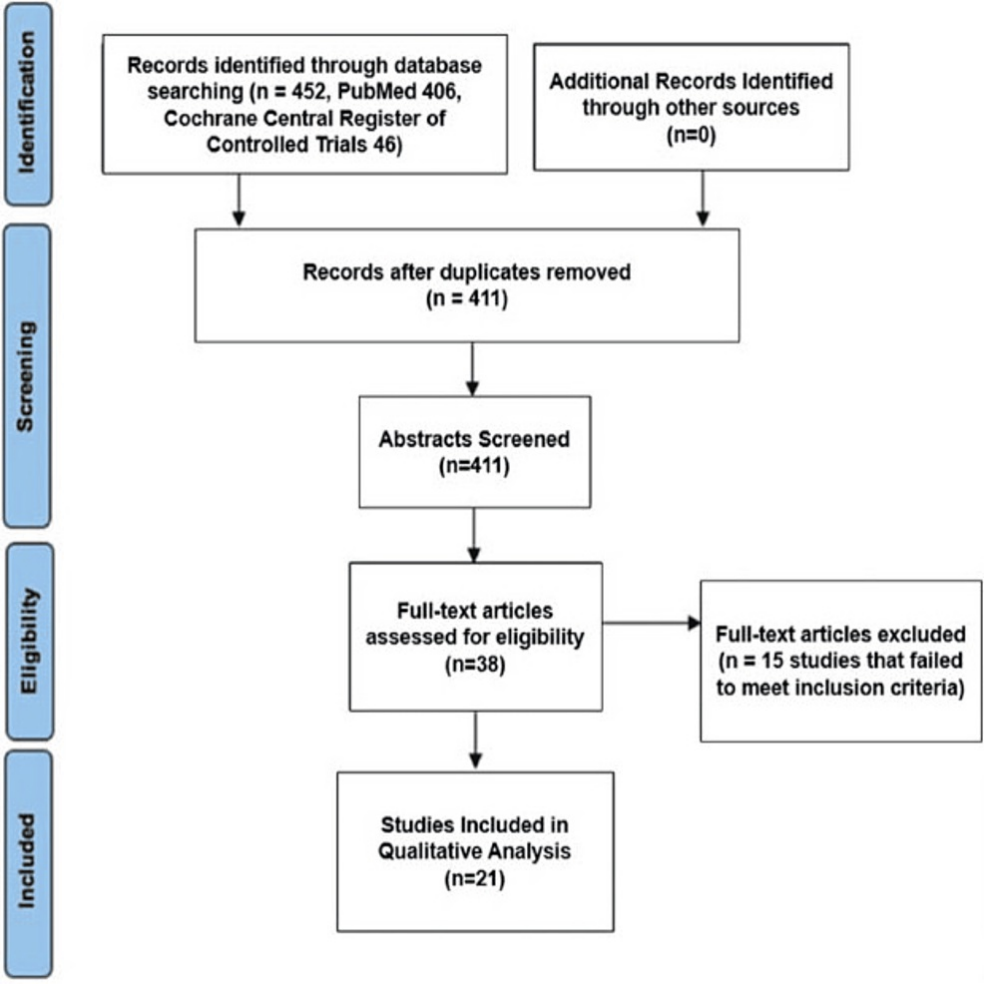
Flow chart depicting literature search procedure and results, following the Preferred Reporting Items for Systematic Reviews and Meta-analyses (PRISMA) guidelines.

The initial title search yielded 452 results, 406 studies from PubMed search, and 46 studies from Cochrane Library. After removal of duplicates, 411 studies were screened by title and abstracts. Full-text reviews were done by the reviewers PB, GG, CI, SS, BJ, AA, and GZ, blinded for each other’s decisions, and evaluated by RV for accuracy. Based on the exclusion criteria, 38 full-text studies were screened, yielding 21 studies that met the inclusion criteria based on the title, abstract content, and discussion of pedicle screw placement using RA or FG. Each study’s references were cross-checked to identify related studies that were originally not found through the search term (n=0) (Figure 1). There was no disagreement among the 10 researchers in the determination of study inclusion and data inclusion.

Data extraction

From the relevant literature, the reviewers collected the following data points when possible: study design (mixed retrospective case-controlled analyses utilizing prospectively collected data, retrospective case-controlled studies, prospective randomized controlled trials), methods (sample size, inclusion and exclusion criteria, study period, statistical analyses), interventions (surgical procedure, therapeutic interventions), and outcomes (further defined below). Statistical analysis and image production were performed using Meta-Essentials version 1.5. [14].

Primary outcome measures

The primary outcome measures include accuracy (success vs failure on the GR classification). Success criteria were defined as GR Grades A & B in one analysis; another analysis defined success criteria as solely GR Grade A [15]. GR Grades are defined as follows: GR Grade A = pedicle screw placed without breaching pedicle cortex, GR Grade B = screw penetrating pedicle cortex less than 2 millimeters (mm), GR Grade C = screw penetrating pedicle cortex 2 - 4 mm, GR Grade D = screw penetrating pedicle cortex 4 - 6 mm, GR Grade E = screw penetrating pedicle cortex more than 6 mm or screw outside the pedicle [16]. GR Grades A and B are typically considered satisfactory results in the literature [17]. Rate ratio was used to compare the rate of success by the robot to the rate of success by FG such that a ratio greater than 1 favors the robot and a ratio less than one favors FG.

Secondary outcomes

Secondary outcomes include facet joint violation (FJV), total case radiation dosage, total case radiation time, total operating room (OR) time, and total case blood loss. FJV was classified per the grading scale used by Babu et al.: Grade 0 = screw not in facet, Grade 1 = screw in lateral facet but not in facet articulation, Grade 2 = penetration of facet articulation by screw, Grade 4 = screw travels within facet articulation [17]. Success criteria were defined as solely Grade 0 in one analysis and as Grade 0 or 1 in another analysis. Rate ratio was used to compare the rate of success by the robot to the rate of success by FG such that a ratio greater than 1 favors the robot and a ratio less than one favors FG.

All other variables (blood loss, total OR time, radiation time, radiation dosage) were analyzed statistically using Hedges’ g analysis. This method was utilized to compare the standardized difference in means of RA and FG values for each paper, Hedges’ g was defined as (Mean value FG - Mean value RA)/pooled standard deviation [18]. Due to the lack of clearly defined events associated with our secondary outcomes, the authors were interested in the comparison between effect sizes. Of note, a negative Hedges’ g denotes a mean FG combined effect size less than the mean RA combined effect size. A positive Hedges’ g thus denotes the opposite. A confidence interval that includes zero is indicative of a lack of statistical significance as the Hedges’ g is a difference of values. 

Results

Primary Outcomes

A total of 21 studies met our inclusion criteria, including six prospective randomized controlled trials, seven retrospective cohort studies, two non-random prospective control studies, one non-random prospective cohort analysis, one prospective case-matched analysis, one matched cohort controlled study, one retrospective matched cohort analysis, and one study with a prospective cohort for RA with a retrospective cohort as the FG control. The types of robots used varied: five used SpineAssist, seven used TiRobot, three used Renaissance, and one study each for ExcelsiusGPS, MazorX ROSA, TINAVI and Orthbot. There were 14 studies determined to have low certainty and seven studies determined to have moderate certainty of evidence. Table 1 summarizes the papers in this segment of the study.

**Table 1:** Accuracy assessments by presence of statistically significant success defined as GR grading of recommendations assessment, with evaluation of evidence quality. GR, Gertzbein-Robbins; Grade, grading of recommendations assessment, development and evaluation; RCT, randomized controlled trial; RA, robot assisted; FG, fluoroscopic guided

Study	Study Type	Robot Used	Total number of patients/screws	Statistically Significant Difference if Success is GR A only?	Statistically Significant Difference if Success is either GR A or B?	Grade quality of evidence
Ringel, Stuer, et al (2012) [19]	Prospective RCT	SpineAssist	RA = 30 / 146 FG = 30 /152	no	no	Moderate
Schizas et al (2012) [20]	Prospective Cohort (non-random)	SpineAssist	RA = 11 / 64 FG = 23 / 64	no	no	Low
Roser et al (2013) [21]	Prospective RCT	SpineAssist	RA = 18 / 72 FG = 10 / 40	no	no	Moderate
Schatlo et al (2014) [8]	Retrospective Cohort	SpineAssist	RA = 55 / 244 FG = 40 / 161	no	no	Low
Lonjon et al (2016) [22]	Prospective case-matched analysis	ROSA	RA = 10 / 46 FG = 10 / 50	no	no	Moderate
Hyun et al (2017) [23]	Prospective RCT	Renaissance	RA = 30 / 130 FG = 30 / 140	no	no	Moderate
Solomiichuk et al (2017) [24]	Matched cohort controlled	SpineAssist	RA = 35 / 192 FG = 35 / 214	no	no	Low
Le, Tian et al (2018) [25]	Retrospective matched-cohort	TiRobot	RA = 20 / 86 FG = 38 / 145	Yes; RA is superior to FG	Yes; RA is superior to FG	Low
Feng et al (2019) [26]	Prospective RCT	TiRobot	RA = 40 / 202 FG = 40 / 225	Yes; RA is superior to FG	no	Moderate
Han, Tian et al (2019) [27]	Prospective RCT	TiRobot	RA = 115 / 532 FG = 119 / 584	Yes; RA is superior to FG	Yes; RA is superior to FG	Moderate
Li, Chen et al (2019) [28]	Prospective RCT	Orthbot	RA = 7 / 32 FG = 10 / 50	no	no	Moderate
Zhang, Xu et al (2019) [29]	Prospective non-random cohort	TiRobot	RA = 50 / 100 FG = 50 / 100	Yes; RA is superior to FG	no	Low
Fayed et al (2020) [30]	Prospective Cohort for RA compared to Retrospective Cohort for FG	ExcelsiusGPS	RA = 20 / 103 FG = 28 / 90	no	no	Low
Cui et al (2021) [31]	Retrospective cohort	Tianji Robot	RA = 23 / 92 FG = 25 / 100	Yes; RA is superior to FG	no	Low
Gao et al (2021) [32]	Retrospective cohort	Renaissance	RA = 18 / 74 FG = 23 / 94	no	no	Low
Katsevman et al (2021) [33]	Retrospective cohort	Mazor X	RA = 17 / 143 FG = 20 / 149	no	no	Low
Zhang, Fan et al (2021) [34]	Retrospective cohort study	Renaissance	RA = 39 / 267 FG = 42 / 288	Yes; RA is superior to FG	no	Low
Su et al (2022) [35]	Prospective nonrandomized, controlled	TiRobot	RA = 28 / 180 FG = 30 / 194	Yes; RA is superior to FG	Yes; RA is superior to FG	Low
Wang et al (2022) [36]	Prospective, nonrandomized, controlled	TiRobot	RA = 61 / 274 FG = 62 / 282	Yes; RA is superior to FG	no	Low
Yan et al (2022) [37]	Retrospective cohort	TiRobot	RA = 40 / 178 FG = 40 / 172	Yes; RA is superior to FG	Yes; RA is superior to FG	Low
Zhang, Zhou et al (2022) [38]	Retrospective cohort	TINAVI Robot	RA = 30 / 158 FG = 14 / 82	Yes; RA is superior to FG	no	Low

All articles were cohort or random-clinical trial study designs. Rate ratios, defined as the proportion of successfully-placed RA pedicle screws divided by the proportion of successfully-place FG pedicle screws, were evaluated to analyze outcomes [19]. When success was defined as GR A or B, the majority of included studies suggested the accuracy of RA pedicle screws was not significantly greater than that of FG pedicle screws (Tables 1, 2). In particular, four out of the 21 included studies suggested RA pedicle screws were significantly more accurate. These articles consisted of two prospective studies (one RCT and one non-randomized controlled trial) and two retrospective cohort studies. The remaining 17 studies suggested there was no significant difference in accuracy between the two approaches. When success was defined as only GR A, there were 10 studies that suggested RA pedicle screws were significantly more accurate than FG pedicle screws. These articles consisted of five retrospective cohort studies, two prospective RCTs, and three prospective non-randomized cohort studies. The remaining 11 studies suggested insignificant differences (Tables 1, 2).

**Table 2:** Rate ratio of accuracy of RA versus FG screws, with success defined as GR grading of recommendations assessment, development and evaluation A or B and GR grading of recommendations assessment, development and evaluation A, respectively. GR, Gertzbein-Robbins; Grade, grading of recommendations assessment, development and evaluation; CI, confidence interval, RA, robot-assisted; FG: fluoroscopic-guided

Study name	Success = GR Grade A or B			Success = GR Grade A		
	Rate Ratio	CI Lower limit	CI Upper limit	Rate Ratio	CI Lower limit	CI Upper limit
Ringel, Stuer (2012) [19]	0.95	0.79	1.15	0.86	0.65	1.13
Schizas (2012) [20]	1.03	0.94	1.13	0.96	0.81	1.14
Roser (2013) [21]	0.99	0.95	1.04	1.01	0.96	1.07
Schatlo (2014) [8]	1.04	0.97	1.11	1.04	0.94	1.14
Lonjon (2016) [22]	1.06	0.96	1.17	1.09	0.93	1.28
Hyun (2017) [23]	1.01	0.99	1.04	1.03	0.98	1.08
Solomiichuk (2017) [24]	1.01	0.93	1.10	1.06	0.92	1.22
Le (2018) [25]	1.10	1.01	1.19	1.30	1.13	1.50
Feng (2019) [26]	1.00	0.99	1.02	1.08	1.03	1.12
Han (2019) [27]	1.06	1.03	1.08	1.11	1.07	1.15
Li (2019) [28]	1.01	0.95	1.08	1.16	0.96	1.40
Zhang, Xu (2019) [29]	1.04	0.98	1.10	1.20	1.03	1.39
Fayed (2020) [30]	0.99	0.96	1.03	0.97	0.92	1.04
Cui et al (2021) [31]	1.00	0.98	1.02	1.11	1.01	1.23
Gao et al (2021) [32]	1.03	0.95	1.12	1.12	0.98	1.27
Katsevman et al (2021) [33]	1.03	0.93	1.15	1.13	0.90	1.42
Zhang, Fan et al (2021) [34]	1.02	0.99	1.04	1.27	1.11	1.45
Su et al (2022) [35]	1.07	1.01	1.12	1.27	1.15	1.41
Wang et al (2022) [36]	1.02	0.99	1.06	1.23	1.12	1.35
Yan et al (2022) [37]	1.10	1.05	1.16	1.20	1.09	1.33
Zhang, Zhou et al (2022) [38]	1.00	0.97	1.04	1.27	1.11	1.45

**Figure 2:**
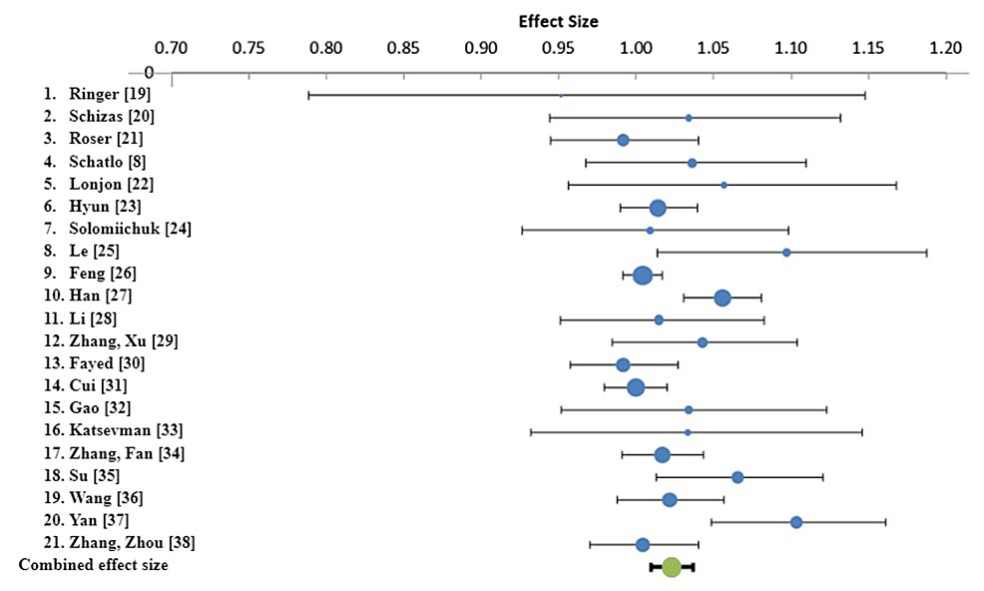
Forest plot for rate ratio for all papers and combined rate ratio. Success is defined as GR grading of recommendations assessment, development and evaluation A or B. GR, Gertzbein-Robbins

**Figure 3:**
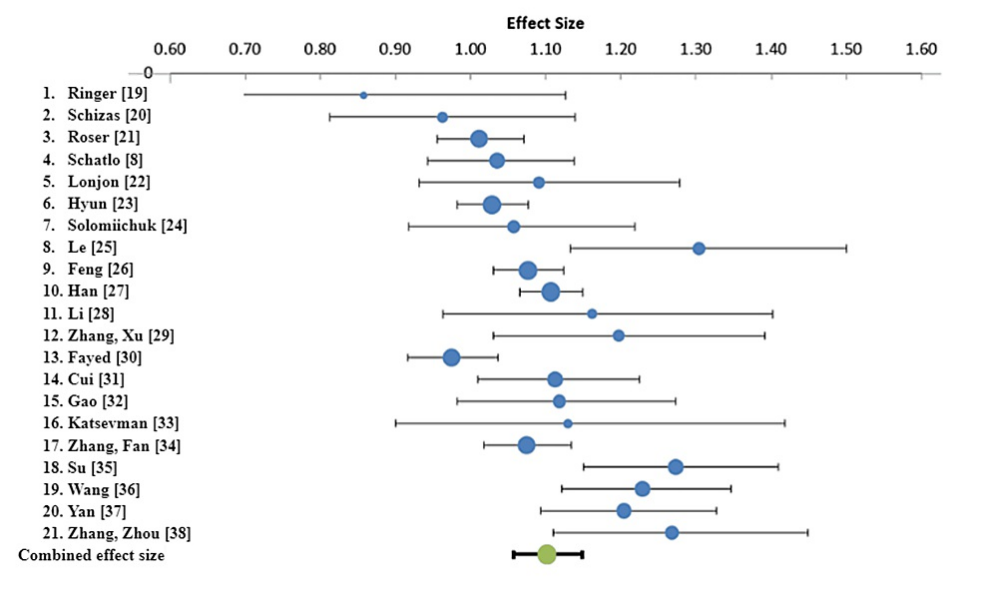
Forest plot for rate ratio for all papers and combined rate ratio. Success is defined as GR grading of recommendations assessment, development and evaluation A. GR, Gertzbein-Robbins

For success defined as GR Grade A or B, RA screws are 1.02 (95% CI: 1.01 - 1.04) times as likely to be successfully placed as their FG counterparts (Table 3). This suggests there is a minimally significant difference between RA and FG screws under this definition of success (Figure 2). There was a larger statistically significant difference between RA and FG screws for success defined as GR Grade A (Figure 3). Under this definition of success, RA screws are 1.10 (95% CI: 1.06 - 1.15) times as likely to be successfully placed as their FG counterparts (Table 3). 

**Table 3:** Overall combined rate ratio for each GR accuracy analysis with confidence intervals. GR, Gertzbein-Robbins; CI, confidence interval

	Success = GR A or B	Success = GR A only
Overall Combined Rate Ratio	1.02	1.10
Lower Limit of CI	1.01	1.06
Upper Limit of CI	1.04	1.15

Secondary Outcomes

There were four studies that included FJV with success defined as either grade 0 or 1, or grade 0 with paper qualities summarized in Table 4. Individual FJV paper rate ratios are included in Table 5. 

**Table 4:** FJV paper qualities. FJV, facet joint violation; Grade, grading of recommendations assessment, development and evaluation; R, robot; FG, fluoroscopic-guided

Study	Study Type	Robot Used	Total number of patients / screws	Statistically Significant Difference in Success is FJV Grade 0 analysis?	Statistically Significant Difference in Success is either FJV Grade 0 or 1 analysis?	Grade quality of evidence
Gao [32]	Retrospective cohort	Renaissance	R = 18 / 36 FG = 23 / 46	yes	no	Low
Zhang, Fan [34]	Retrospective cohort	Renaissance	R = 39 / 78 FG = 42 / 82	yes	no	Low
Li [39]	Retrospective cohort	TiRobot	R = 37 / 172 FG = 44 / 204	yes	no	Low
Wang [36]	Prospective cohort, nonrandomized	TiRobot	R = 61 / 274 FG = 62 / 282	yes	yes	Low

**Table 5:** FJV paper rate ratios FJV, facet joint violation; Grade, grading of recommendations assessment, development and evaluation; CI, confidence interval

Study name	Success = FJV Grade 0 or 1			Success = FJV Grade 0 only		
	Rate Ratio	CI Lower limit	CI Upper limit	Rate Ratio	CI Lower limit	CI Upper limit
Gao [32]	1.09	0.97	1.23	1.32	1.08	1.61
Zhang, Fan [34]	1.01	0.97	1.06	1.24	1.08	1.42
Li [39]	1.08	1.03	1.13	1.23	1.12	1.35
Wang [36]	1.07	0.97	1.17	1.23	1.01	1.50

With success defined as FJV Grades of 0 or 1, no significant differences were found between RA and FG pedicle screws (Combined Rate Ratio: 1.05, 95% CI: 0.99-1.12). Of the four studies that met the inclusion criteria for FJV, only one study showed significant support for RA pedicle screws based on these criteria. However, with success defined as FJV Grade 0 only, RA screws were 1.24 (95% CI: 1.19-1.29) times more likely to be successfully placed as compared to their FG counterparts. All four included studies individually demonstrated statistical significance in favor of RA pedicle screw placement when success is defined solely as FJV Grade 0 (Table 6). 

**Table 6:** Overall combined rate ratio for each GR accuracy analysis with confidence intervals. GR, Gertzbein-Robbins; FJV, facet joint violation; Grade, grading of recommendations assessment, development and evaluation; CI, confidence interval

	Success= FJV Grade 0 or 1	Success = FJV Grade 0 only
Overall Combined Rate Ratio (n=355 screws)	1.05	1.24
Lower Limit of CI	0.99	1.19
Upper Limit of CI	1.12	1.29

There were 20 studies included for the evaluation of other secondary outcomes such as blood loss, total OR time, total case radiation dosage, and total case radiation time. The individual quality and characteristics of the 20 studies are summarized in Table 7.

**Table 7:** Nominal variables paper qualities. An ‘x’ denotes papers that have enough data on the respective nominal variable but without statistically significant differences. An R denotes favoring the robot. An F denotes favoring FG. OR, operating room; Grade, grading of recommendations assessment, development and evaluation; R, robot; FG, fluoroscopic-guided

Study	Study Type	Robot Used	Total number of patients / screws	Blood Loss, case total	Total OR time	Total case radiation dosage	Total case radiation time	Grade quality of evidence	
Ringel [19]	Prospective controlled	Spineassist	R = 30 / 146 FG = 30 / 152		x		x	Moderate	
Schizas [20]	Prospective controlled	Spineassist	R = 11 / 64 FG = 23 / 64			x	x	Low	
Roser [21]	Randomized clinical trial	Spineassist	R = 18 / 72 FG = 10 / 40				R	Moderate	
Schatlo [8]	Retrospective cohort	Spineassist	R = 55 / 244 FG = 40 / 161		x			Low	
Lonjon [22]	Prospective controlled cohort	ROSA	R = 10 / 36 FG = 10 / 50		F		F	Low	
Hyun [23]	Randomized clinical trial	Renaissance	R = 30 / 130 FG = 30 / 140		x	R	R	Moderate	
Solomiichuk [24]	Prospective matched controlled	Spineassist	R = 35 / 192 FG = 35 / 214		x		x	Low	
Le [25]	Retrospective cohort	TiRobot	R = 20 / 86 FG = 38 / 145		F	R	F	Low	
Feng [26]	Randomized clinical trial	TiRobot	R = 40 / 202 FG = 40 / 225			R		Moderate	
Han [27]	Randomized Clinical Trial	TiRobot	R = 115 / 532 FG = 119 / 584	R	x	R	x	Moderate	
Li [28]	Randomized Clinical Trial	Orthbot	R = 7 / 32 FG = 10 / 50	x			R	Moderate	
Zhang, Xu [29]	Prospective nonrandomized cohort	TiRobot	R = 50 / 100 FG = 50 / 100	R	F	R	x	Low	
Fayed [30]	Prospective Cohort for RA compared to Retrospective Cohort for FG	ExcelsiusGPS	RA = 20 / 103 FG = 28 / 90					Low	
Cui [31]	Retrospective controlled	Tianji	R = 23 / 92 FG = 25 / 100	R	F			Low	
Gao [32]	Retrospective cohort controlled	Renaissance	R = 18 / 74 FG = 23 / 94				R	Low	
Katsevman [33]	Retrospective cohort	Mazor X Robot	R = 17 / 143 FG = 20 / 149					Low	
Zhang, Fan [34]	Retrospective cohort	Renaissance	R = 39 / 267 FG = 42 / 288	R			R	Low	
Li [39]	Retrospective controlled	TiRobot	R = 37 / 172 FG = 44 / 204	R			R	Moderate	
Su [35]	Prospective nonrandomized	TiRobot	R = 28 / 180 FG = 30 / 194	F	F	R	R	Low	
Wang [36]	Prospective cohort	TiRobot	R = 61 / 274 FG = 62 / 282		F		R	Low	
Yan [37]	Retrospective cohort	TiRobot	R = 40 / 178 FG = 40 / 172	x	x			Low	
Zhang, Zhou [38]	Retrospective controlled	TINAVI	R = 30 / 158 FG = 14 / 82	x				Low	

Overall, RA pedicle screw placement was associated with no significant difference in blood loss (Hedges' g: 1.16, 95% CI: -0.75 to 3.06) (Table 8). FG was also associated with significantly shorter OR time (Hedges' g: -1.03, 95% CI: -1.76 to -0.31). There was no significant difference (Hedges' g: -0.34, 95% CI: -1.22 to 0.53) in case radiation time between RA and FG groups. However, total case radiation dosage was significantly higher in the FG group (Hedges' g: 1.61, 95% CI: 1.11 to 2.10).

**Table 8:** Table of combined Hedges’ g and confidence intervals for all nominal variables. CI, confidence interval; OR, operating room

Metric	Number of Studies	Number of Total Patients (n)	Overall Combined Hedges’ g	Lower Limit CI	Upper Limit CI
Blood loss, total case	7	619	1.16	-0.75	3.06
Total OR time	10	866	-1.03	-1.76	-0.31
Total case radiation dosage	6	590	1.61	1.11	2.1
Total case radiation time	6	643	-0.34	-1.22	0.53

Discussion

The use of robotics for pedicle screw placement is the culmination of multiple technologies and advancements that have been building upon each other for decades. The central question surrounding the implementation of these robots is whether they lead to significantly more accurate placement of pedicle screws than conventional techniques. Historically, the literature has shown variable accuracy for FG without navigation [9]. Computer navigation systems were introduced to pedicle screw placement surgery with the goals of improving accuracy and reducing the need for screw revision [40]. In a large meta-analysis spanning 130 studies and over 37,000 pedicle screws, Kosmopoulos and Schizas found that navigation did indeed lead to improved accuracy for pedicle screw placement, with the middle two quartiles of studies ranging between 75% and 95% accuracy for screws placed without navigation and between 85% and 97% accuracy with navigation [6]. In another meta-analysis performed by Verma in 2010 comparing navigational to non-navigational techniques, an accuracy rate of 93.3% was found for screws placed with navigation against 84.7% accuracy for non-navigational techniques [41]. However, these differences were deemed statistically insignificant by the authors.

The Mazor Spineassist was the first robot to be studied for accuracy in pedicle screw placement and is still perhaps the most extensively studied robot for minimally invasive spine surgery to date [9]. In an early case series of 14 patients, Sukovich reported a 93% success rate using the Spineassist and a 96% accuracy rate as defined by screws placed within 1mm of their planned trajectory [42]. In a multicenter retrospective study by Devito of 3,271 pedicle screws in 635 cases, 646 screws were evaluated postoperatively by CT scan [43]. Of the screws imaged postoperatively, 98% were found to be clinically acceptable with 89% placed completely within the pedicle. Additionally, the authors found that in the RA cases, no patients experienced permanent neurologic deficits compared to the 0.6 - 5% reported in the literature for conventional techniques [44-46].

In one prospective randomized controlled study from Ringel, FG technique demonstrated a higher accuracy rate than the Spineassist [19]. In this study, the FG placement group had an accuracy of 93% while the Spineassist cohort had clinically acceptable positions in 85% of cases. The authors noted two possible reasons for this result. First, insufficient stable attachment of the robot to the patient's spine, and second the slipping of the cannula relative to the bone or so-called “skiving”. To date, this is the only RCT that found the RA approach to be inferior [20]. In the opinion of one of the authors, with advancements in technology, skiving has been minimized due to RA and high-speed drills, which allows for higher accuracies of screw placements. Ultimately, improvements in technology, such as RA surgeries, skiving, and radiation are expected to decrease [19]. This builds upon the facts provided throughout the literature review in this meta-analysis.

We conducted a meta-analysis in order to assess accuracy and radiation exposures in pedicle screws placed in FG versus RA. According to our findings, screws placed with RA screws are significantly more accurate for GR Grade A but not for the clinically acceptable criteria of grade A+B. These findings suggest that the use of robots may mainly increase intrapedicular fidelity of screws that have not breached the pedicular cortex. Our results confirm the findings of similar meta-analyses that have been published; Fu et al. described in their meta-analysis that RA screws are 2.43 times as likely as FH screws to achieve Grade A GR classification [47]. 

The pronounced effect of pedicular screw placement accuracy in GR grade A when compared to grades A+B in RA vs FG techniques may be due to the inherent stability offered by the robot. RA pedicle screw insertion is associated with a low rate of cortical bone breaches due to the pre-planned trajectory of the arm’s movements and screw insertion, as well as the tools used to help guide the proper placement [39]. The authors hypothesize that utilizing tools such as the cannula prior to screw insertion with the robot can help guide the pedicle screw in the appropriate trajectory and minimizes the risk of deviation. In contrast, when performing pedicle screw insertion via the FG technique, tools such as this are not utilized and may result in movement of the screw about the reference frame during placement [48,49]. In FG cases, the accuracy of screw placement relies on the surgeon’s experience and skill. When considering both techniques, RA screw insertion may have the advantage of consistency and steadiness during the insertion process as well as minimizing human error that can come from weakness or fatigue experienced in the FG technique. Overall, these factors may contribute to decreasing the risk of cortical breach in RA screw placement. 

FJV is also a major concern in pedicle screw placement. Our findings suggest that RA screws and FG screws both have similar rates of achieving Grade 0 or Grade 1 (screw does not encroach upon the facet joint or screw was in lateral facet but does not enter articular facet). However, RA screws were significantly more likely to achieve Grade 0 in comparison to FG screws. Ours is one of the first meta-analyses to our knowledge to directly compare RA screws to FG screws in FJV grading.

Blood loss was statistically similar between RA and FG cases. This finding is not in agreement with Fu et al.’s meta-analysis, which described RA cases to have a significantly lower blood loss than FH cases [47]. However, Fu et al.’s meta-analysis strictly compared conventional FH techniques to robotic techniques while our study uses FG cases as the control, which may have yielded discrepancies in the data included in the studies.

Radiation exposure is also a concern for members of the surgical team. Our study looked at both radiation time per case and radiation dosage per case. Our analysis of radiation exposure to the surgeon with RA looked at seconds per surgery, which differs from previous meta-analyses which averaged numbers from studies examining radiation per screw and radiation per surgery as mentioned in a meta-analysis by Li et al. [50]. Overall, while there was a statistically insignificant difference in radiation time per case, there was a statistically significant decrease in radiation dose per case with RA surgery. It would reason that radiation exposure is inversely related to the surgeon's experience operating the robot. As surgical robots become more widespread, perhaps radiation exposure will decline during these surgeries.

Total OR time was found to be significantly shorter for FG cases than for RA cases. This is in agreement with anecdotal evidence; however, there is conflicting evidence in the literature. Fu et al. describe in their meta-analysis that there was no significant difference between RA cases and FH cases [47]. On the contrary, Li et al. described in their meta-analysis that robotic cases were significantly longer than FH cases [51]. Again, however, this meta-analysis differs from our study in that we omitted true FH cases that did not use intraoperative FG. The conflicting results in several studies in the literature suggest that additional high-powered prospective studies are required in order to better characterize the differences, if any, between RA cases and FG cases as well as FH cases. Overall, our findings are in line with previous meta-analyses done by Gao et al. [32] and Li et al. [39]; these were smaller studies based only on RCTs and primarily used FG technique as the control group. 

One potential weakness of our meta-analysis is due to the fact many of our studies used in the analysis are cohort studies which are by nature lower quality studies than randomized control trials. This was primarily due to the paucity of large randomized control trials that have been conducted in this specific area comparing the two approaches. Currently, while many studies support the use of RA techniques, there also exists evidence suggesting inferiority to FG [19,27]. As there continue to be more RCTs however, future studies could involve larger, higher-quality meta-analyses that stratify subtypes of robotic surgeries to compare their accuracy against different FH and navigation approaches. 

## Conclusions

Since the advent of robotic assistance in pedicle screw placement surgeries, the practicing surgeon has been faced with the decision to rely on tried-and-true methods in fluoroscopically guided pedicle screw placement or utilize a robot’s operating mechanics in an attempt to improve outcomes. Our meta-analysis presents low-quality evidence suggesting that robotically assisted pedicle screws have improved accuracy with statistical significance compared to fluoroscopically guided pedicle screws if success is defined solely as Gertzbein and Robbins classification A. Robotically assisted pedicle screws were also found to be marginally better when defined as the traditional Gertzbein and Robbins classification of A or B. Furthermore, we present low-quality evidence suggesting that robotically assisted pedicle screw surgeries utilize less radiation dosage exposure than fluoroscopically guided surgeries. Robotic cases were found to be significantly longer than fluoroscopically guided cases. Future standardized and well-powered prospective studies are necessary to more clearly assess the efficacy of robotic assistance in pedicle screw placement. 
